# Correlation Between Plasma CircRNA-089763 and Postoperative Cognitive Dysfunction in Elderly Patients Undergoing Non-cardiac Surgery

**DOI:** 10.3389/fnbeh.2020.587715

**Published:** 2020-10-06

**Authors:** Hongli Zhou, Fuyu Li, Wanlin Ye, Maozhou Wang, Xian Zhou, Jianguo Feng, Li Liu, Xiaobin Wang

**Affiliations:** ^1^Department of Anesthesiology, The Affiliated Hospital of Southwest Medical University, Luzhou, China; ^2^Heart Center and Beijing Key Laboratory of Hypertension, Beijing Chaoyang Hospital, Capital Medical University, Beijing, China; ^3^Department of Internal Medicine, The Affiliated Hospital of Southwest Medical University, Luzhou, China; ^4^Laboratory of Anesthesiology, The Affiliated Hospital of Southwest Medical University, Luzhou, China

**Keywords:** circRNAs, postoperative cognitive dysfunction, qRT-PCR, *Z* score method, elderly patients

## Abstract

In our previous experiment, we found that there were abnormal levels of circRNA-089763 in the plasma exosomes of patients with postoperative cognitive dysfunction (POCD) after cardiac surgery. Therefore, the aim of this study was to further investigate the relationship between plasma circRNA-089763 level and POCD in elderly patients after non-cardiac surgery. A prospective cohort study was conducted to select elderly patients undergoing elective non-cardiac surgery. A total of 72 patients were enrolled in this study, and cognitive functions were assessed 1 day before and 3 days after surgery by a series of neuropsychological measurements. Next, patients were divided into POCD and non-POCD (NPOCD) groups according to the *Z* score method. Blood was collected the day before and 3 days after surgery, and the plasma circRNA-089763 level was detected by quantitative real-time polymerase chain reaction (qRT-PCR). Then, the difference and correlation in plasma circRNA-089763 levels between the POCD and NPOCD groups were analyzed. On the third day after surgery, the incidence of POCD was 30.56%. The relative level of circRNA-089763 in the POCD group was 2.41 times higher than that in the NPOCD group (*t* = 4.711, *p* < 0.001), patients in POCD group had higher age (*t* = 5.971, *p* < 0.001), higher American Society of Anesthesiologists classification (χ^2^ = 14.726, *p* < 0.001), less years of education (*t* = 2.449, *p* = 0.017), more intraoperative blood loss (*t* = 3.196, *p* = 0.002), and higher visual analog scale (VAS) scores (*t* = 10.45, *p* < 0.001). The binary logistic regression analysis showed that the circRNA-089763 level, age, and intraoperative blood loss were independently associated with POCD (OR: 2.75, 95% CI: 1.261–5.999, *p* = 0.011; OR: 1.32, 95% CI: 1.114–1.565, *p* = 0.001; OR: 1.017, 95% CI: 1.004–1.03, *p* = 0.011). These results demonstrated that the circRNA-089763 plasma level was related to POCD after non-cardiac surgery in elderly patients.

## Introduction

Circular RNAs (CircRNAs) are a novel type of non-coding RNA with a closed loop structure (Li et al., 2018). CircRNA is widely present in the eukaryotic transcriptome and can regulate target gene expression by the competitive endogenous RNA (ceRNA) mechanism (Tay et al., 2014). *In vivo*, circRNAs are predominantly transported in the form of exosomes, which can enter the blood circulation through the blood–brain barrier (Li et al., 2015).

Recent studies have found that circRNAs may play crucial roles in neurological diseases, such as Alzheimer’s disease (AD) (Zhao et al., 2016; Sekar et al., 2018; Wang et al., 2018; Dube et al., 2019). Additionally, Zhang et al. (2017) characterized circRNA-associated ceRNA networks in senescence-accelerated mouse prone 8 brain and found that these networks could affect the diagnosis and therapy of AD in the near future. The dysfunction of the circRNA-miRNA-mRNA regulatory system seems to represent another important aspect of epigenetic control of the human central nervous system’s pathogenic gene expression program. Moreover, postoperative cognitive dysfunction (POCD) and AD have similar pathogenesis (Hu et al., 2010; Hua et al., 2014).

POCD is a common complication of neurological diseases in elderly patients that severely affects quality of life (Rundshagen, 2014). Therefore, early detection, diagnosis, and intervention in POCD will effectively improve the quality of life of surgical patients. However, the mechanism of POCD is not yet clear, and unified clinical criteria to diagnose POCD are still lacking. Therefore, it is imperative to find reliable and convenient clinical biomarkers.

In clinical work, peripheral blood samples are easy to collect compared with other specimens, such as cerebrospinal fluid (CSF) and brain tissues. Studies have found that brain tissues can release exosomes, which can carry non-coding RNAs (ncRNAs, such as microRNAs, long stranded non-coding RNAs, and circRNAs) and enter the blood circulation through the blood–brain barrier. In our previous experiment, we found that there were abnormal levels of circRNA-089763 in plasma exosomes of POCD patients after cardiac surgery (Wang et al., 2019). However, we found that it was difficult to collect and extract exosomes, and the level of total plasma (not only plasma exosomes) circRNA-089763 in non-cardiac POCD patients is still unknown. Therefore, this study aims to investigate whether the level of circRNA-089763 in the plasma of elderly patients undergoing non-cardiac surgery exhibits the same changes, to analyze the interaction and to provide novel insights about the underlying mechanisms of POCD.

## Materials and Methods

### Subjects

**FIGURE 1:**
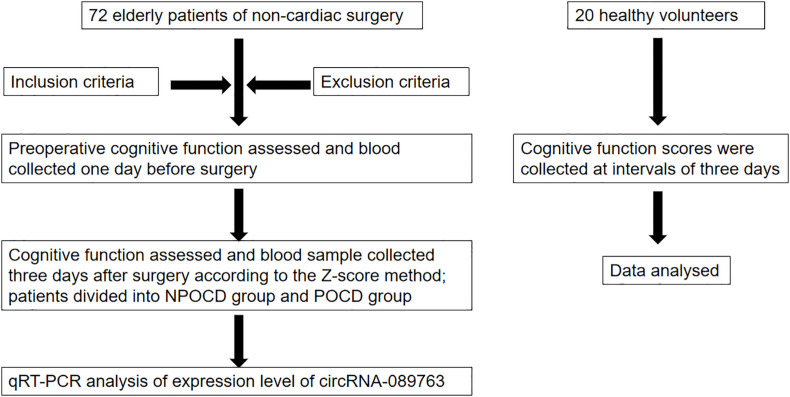
Experiment flow chart.

The protocol was reviewed and approved by the Ethics Committee of Clinical Trials in the Affiliated Hospital of Southwest Medical University (Approval#: 20180306038; Trial registration: ChiCTR1800016435, registered 1 June 2018). Written informed consent was obtained from the patients or their relatives before study enrollment. Patients scheduled to undergo general anesthesia for non-cardiac surgery in the Affiliated Hospital of Southwest Medical University from June 2018 to June 2019 were enrolled in this study. Blood samples were taken from patients undergoing surgery. To exclude the role of learning memory and increase the credibility of the POCD diagnosis, 20 healthy volunteers were recruited as the control group, and cognitive function assessment was completed only for the cognitive function scales. The experimental technology route is shown in Figure 1.

Inclusion criteria included the following: age ≥ 65 years old, American Society of Anesthesiologists (ASA) grade I–III, patient consent, estimated operation time ≥ 2 h. Patients were excluded from study participation if they met any of the following criteria: cognitive impairment characterized by Mini-Mental State Examination (MMSE) score < 24 before surgery, mental disorders, history of dementia, severe organ dysfunction (respiratory, circulatory, or other system dysfunction), refusal to phlebotomize, cognitive function scale assessment difficulty (low-compliance patients, severe hearing impairment, visual impairment, disability of reading and understanding), cardiovascular and cerebrovascular accidents occurring in the past 6 months, serious complications after surgery, and blood samples not meeting test requirements.

### Anesthesia Protocol

Vital signs were monitored once patients came into the operating room, and the patient received oxygen inhalation (6 L/min). Induction of anesthesia was as follows: penehyclidine hydrochloride (0.01 mg/kg), propofol (1.5–2.5 mg/kg), or etomidate (0.2–0.4 mg/kg), sufentanil (0.2–0.5 mg/kg), and cisatracurium (0.1–0.2 mg/kg). After anesthesia induction and tracheal intubation, anesthesia maintenance included remifentanil (0.1–0.2 μg/kg⋅min), cisatracurium (1–2 μg/kg⋅min), and sevoflurane (1.5–3%). Effective circulatory blood pressure, respiratory index, and appropriate anesthesia depth were maintained during the operation.

### Neuropsychological Assessments and POCD Judgment Methods

Neuropsychological tests were primarily used to evaluate attention and executive ability, memory and learning ability, visual spatial awareness, and language fluency. The series of scales, including MMSE, Color Trail Test (CTT), Digital Span Test (DST), Clock Drawing Test (CDT), and Verbal Fluency Test (VFT), were performed the day before surgery and third day after surgery. At the same time, 20 healthy volunteers without operation (age ≥ 65 years old, matched for education level and gender) were selected as the control group to determine a normal reference value of cognitive functions (cognitive functions were assessed twice at 3-day intervals).

These tests were administered by one experienced research staff member who received one-on-one training and finished 10 independent assessments before the start of the study. She was blinded to the surgical procedure and blood samples results. Patients were first screened with MMSE to exclude severe cognitive impairment. The MMSE assesses a wide range of domains, including attention, language, memory, orientation, and visuospatial proficiency; it requires about 6 to 10 min to administer. CTT is frequently utilized to assess the capability of attention conversion; numbers were placed in circles with the background colors of yellow or red, and subjects were required to connect the numbers in numerical order. Completion time was recorded as the index of attention conversion (the longer the time, the lower the efficiency of attention conversion). DST was used to assess the ability of focusing the transient memory, mind, and anti-jamming; the subjects were asked to repeat digits immediately after the investigator; the number of digits increased, and the highest number was taken as the score. The CDT includes an already predrawn circle. The most common administration instructions are “Please draw a clock face, placing all the numbers on it. Now set the time to 10 past 11.” VFT evaluates the ability to form and fluently utter words. The test consists of three parts. The first two parts consist in listing as many words as possible in 60 s (usually names of objects). In the third part the objective is to list in 60 s as many words as possible that belong to a given phonetic category. In the present study these categories were names of animals (category 1), names of sharp objects (category 2), and words beginning with the letter J (category 3). The result of the test is the number of correct words listed for each of the categories. Participants were given the following instructions: “Now I want to see how many different animals you can name. You will have 60 s. When I say, ‘Begin,’ say the animal names as fast as you can.” Fluency was the total number of animals named in 60 s.

According to the International Study of Postoperative Cognitive Dysfunction guidelines (Moller et al., 1998), the “*Z*-score method” was applied to determine whether enrolled patients had POCD. Formula: *Z* = (Δ*X* −Δ*XC*)/SD_Δ*XC*_. Δ*X* was the difference between preoperative and postoperative cognitive test scores in the operation group, while Δ*XC* referred to the average value of the difference in the same scale in the control group. SD_(Δ*XC)*_ was the standard deviation of the difference between the same scale in the control group. The patients were considered to have POCD when there were at least two |*Z*| scales greater than 1.96, and thus were divided into the POCD group and non-POCD (NPOCD) group.

### Data Collection

Demographic and intraoperative data were collected, such as age, education time, weight, height, body mass index (BMI), anesthesia methods, operative time, intraoperative blood loss, intraoperative rehydration, cognitive function assessment scores of patients at 1 day before and 3 days after operation, and VAS on the third day after surgery. All fasting peripheral venous blood specimens were collected in anticoagulant tubes with EDTA-K2 1 day before surgery and 3 days after surgery. After mixing the specimens upside down, they were placed at room temperature for 1–2 h and then centrifuged at 4°C at 3,000 rpm for 15 min. The upper plasma sample was gently pipetted with a RNase-Free pipette, dispensed in a 1.5 ml RNase-Free cryotube, and stored at −80°C until further analysis.

### Plasma CircRNA Detection

In accordance with the manufacturer’s instructions, the preserved plasma was thawed at room temperature. Total RNA was extracted by lysate MZA reagent (TIANGEN, China), after which 900 μl of lysate MZA reagent was added per 200 μl of plasma, and from each specimen, a total 800 μl of plasma was extracted. Next, the NanoDrop-2000 was used to detect the quality of the RNA sample, 260/280 optical density ratios > 1.80 (good quality). Total RNA content ≥ 1,000 ng indicated that the quality was acceptable for further experiments. Among the acceptable plasma samples, the total RNA concentration of three patients in the POCD group and four patients in the NPOCD group was too low, and thus their samples were excluded, leaving 46 patients in the NPOCD group and 19 patients in the POCD group for circRNA-089763 analysis. The random primer method was used to reverse transcribe cDNA. Using cDNA as a template, quantitative real-time polymerase chain reaction (qRT-PCR) was conducted according to the instructions of the SYBR Green PCR Master Mix Kit (QIAGEN). Reaction system: 2xSYBR Green PCR Master Mix 10 μl, QN ROX Reference Dye 2 μl, PCR Forward Primer (10 μM) 1 μl, PCR Reverse Primer (10 μM) 1 μl, 3 μl of cDNA solution, and enough RNase-free water to reach a total of 20 μl. Reaction conditions: 95.0°C 10 min 1 cycle; 95.0°C 15 s, 60.0°C 1 min, 50 cycles; 95.0°C 15 s, 60.0°C 1 min, 1 cycle; and 60.0°C 30 s, 1 cycle. Taking β-actin as an internal reference, the Delta-delta Ct method, F (Fold change) = 2^–△△*Ct*^, was analyzed for circRNA expression levels. β-actin and circRNA-089763 primer were designed by primer 5.0 software and synthesized by the Beijing Genomics Institute (Table 1).

**TABLE 1:** β-actin and circRNA-089763 primers.

Gene	Primer sequence	Tm (°C)	GC (%)
β-actin	F:5′CTCTTCCAGCCTTCCTTCCT3′	57.45	55
	R:5′AGCACTGTGTTGGCGTACAG3′	57.45	55
circRNA-089763	F:5′GGTGATGAGGAATAGTGTAAGG3′	56.26	45
	R:5′ACCTCCATCATCACCTCAACC3′	57.80	52

### Statistical Methods

Statistical analyses were conducted using SPSS Statistics 17.0 (IBM corp.; Armonk, NY, United States). Patients were split into POCD and NPOCD groups according to the “*Z*-score method.” Normally distributed continuous data were expressed in the form of mean ± standard deviation, and Student’s *t*-test (unpaired *t*-test with Welch’s correction) was used for data analysis. The categorical data were expressed as frequencies or percentages and analyzed by Pearson chi-square (χ^2^) test or Fisher’s exact probability test. Binary logistic regression analysis was used to analyze the influence factors for POCD. All statistical tests were two-tailed, and *p* < 0.05 indicated that the difference was statistically significant.

## Results

### Patient Information

In this study, 72 patients met the inclusion criteria and participated in the research. Meanwhile, 20 healthy volunteers who did not undergo operation were recruited as the control group. As shown in Table 2, there were no statistical differences in the basic data (gender, age, years of education, and BMI) between the control and non-cardiac elderly surgery groups (test group) (unpaired *t*-test with Welch’s correction or χ^2^ test).

**TABLE 2:** The demographics data of the control and test groups.

Demographics of patients	Control group (*n* = 20)	Test group (*n* = 72)	*P-*value
Gender (male/female)	13/7	43/29	0.669
Age (years)	70.45 ± 3.15	71.15 ± 5.95	0.853
Years of education	6.55 ± 2.38	6.53 ± 2.75	0.753
BMI (kg⋅m^–2^)	23.98 ± 2.95	24.31 ± 2.94	0.355
MMSE scores 1 day before surgery	28.70 ± 1.03	26.90 ± 1.54	0.051
CTT scores 1 day before surgery	35.90 ± 3.46	36.28 ± 5.49	0.937
DST scores 1 day before surgery	7.76 ± 1.64	7.71 ± 1.46	0.984
CDT scores 1 day before surgery	3.62 ± 0.50	3.19 ± 0.57	0.228
VFT scores 1 day before surgery	26.81 ± 3.70	28.82 ± 4.22	0.054

**BMI, body mass index; MMSE, Mini-Mental State Examination; CTT, Color Trail Test; DST, Digit Span Test; CDT, Clock Drawing Test; VFT, Verbal Fluency Test.**

The cognitive function scores of volunteers at intervals of 3 days and SD_(Δ*XC)*_ are shown in Table 3. In the volunteers’ two VFT tests, the score of the second test was significantly increased compared with that of the first test (*t* = 3.376, *p* = 0.003). According to “*Z*-score method” (Moller et al., 1998), POCD was defined as the presence of at least two cognitive function scales of |*Z*| ≥ 1.96. As shown in Table 4, there were 22 patients meeting this definition, yielding a POCD incidence of 30.56%.

**TABLE 3:** The cognitive function scores of volunteers.

Cognitive assessment	Volunteers as control group (*n* = 20)		
	1st assessment	2nd assessment	SD_(Δ*XC)*_
MMSE	28.70 ± 1.03	28.35 ± 1.35	0.51
CTT	35.90 ± 3.46	34.76 ± 4.60	1.15
DST	7.76 ± 1.64	8.14 ± 1.11	1.04
CDT	3.62 ± 0.50	3.48 ± 0.51	0.43
VFT	26.81 ± 3.79	29.14 ± 3.90*	1.03

*vs. 1st assessment*, *p < 0.05.

**TABLE 4:** The cognitive function scores in NPOCD and POCD groups.

Cognitive assessments	NPOCD (*n* = 50)		POCD (*n* = 22)	
	1 day before surgery	3 day after surgery	1 day before surgery	3 day after surgery
MMSE	26.98 ± 1.71	25.62 ± 1.60*	26.73 ± 1.12	23.09 ± 1.44^∗#^
CTT	36.10 ± 5.85	40.84 ± 6.93*	36.68 ± 4.82	43.55 ± 6.16^∗#^
DST	7.78 ± 1.38	7.32 ± 1.27*	7.55 ± 1.68	6.27 ± 1.08^∗#^
CDT	3.12 ± 0.59	2.70 ± 0.86*	3.36 ± 0.49	1.95 ± 0.72^∗#^
VFT	28.94 ± 4.18	27.78 ± 4.01	28.36 ± 4.24	21.05 ± 3.67^∗#^

vs 1 day before surgery, *p < 0.05, vs. NPOCD group, ^#^p < 0.05.

As illustrated in Table 5, the main demographic characteristics and intraoperative conditions of the POCD and NPOCD groups were summarized, and there were significantly older ages (*t* = 5.971, *p* < 0.001), less years of education (*t* = 2.449, *p* = 0.017), higher ASA grades (χ***^2^*** = 14.726, *p* < 0.001), more intraoperative blood loss (*t* = 3.196, *p* = 0.002), and higher VAS scores (*t* = 10.45, *p* < 0.001) in the POCD group than in the NPOCD group. No differences in gender, BMI, anesthesia time, surgical time, or intraoperative fluid volume were observed between the POCD and NPOCD groups (*p* > 0.05).

**TABLE 5:** Basic information of NPOCD and POCD groups.

Characteristics of patients	NPOCD group (*n* = 50)	POCD group (*n* = 22)	*t*/χ^2^-value	*P-*value
Gender (male/female)	29/21	14/8	0.035	0.851
Age (years)	68.86 ± 4.52	76.36 ± 5.72	5.971	< 0.001
Years of education (years)	7.04 ± 2.81	5.36 ± 2.32	2.449	0.017
BMI (kg⋅m^–2^)	24.00 ± 2.88	24.99 ± 3.11	1.312	0.194
ASA (I/II/III)	18/23/9	2/5/15	14.726	< 0.001
Anesthesia time (min)	217.90 ± 37.93	222.27 ± 35.98	0.458	0.649
Surgical time (min)	189.60 ± 36.01	191.41 ± 31.42	0.204	0.839
Intraoperative blood loss (ml)	228.80 ± 81.68	296.59 ± 85.71	3.196	0.002
Intraoperative fluid volume (ml)	1579.00 ± 340.25	1488.64 ± 253.51	1.115	0.269
VAS scores at postoperative 3 day	1.88 ± 0.79	4.14 ± 0.92	10.45	< 0.001

### CircRNA Expression Level

**FIGURE 2:**
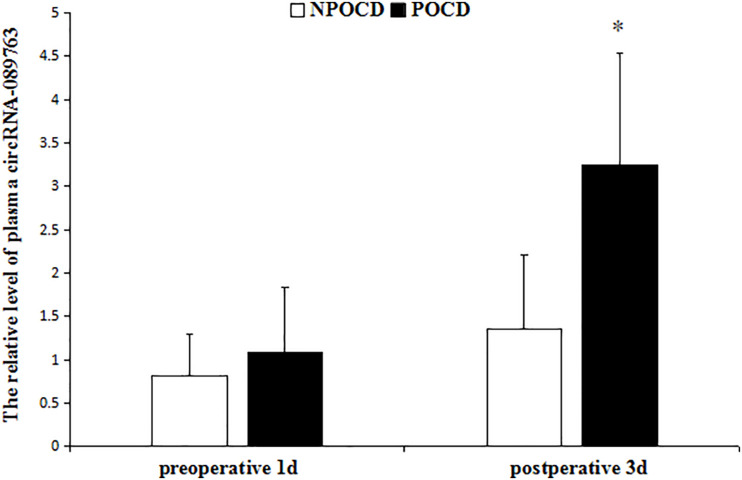
The relative level of plasma circRNA-089763 in the two groups. The data are shown as the means ± SD. vs NPOCD in postoperative 3d, *P* < 0.001 in the legend of Figure 2.

As shown in Figure 2, there was no significant difference in relative circRNA-089763 plasma level 1 day before the operation (*t* = 1.772, *p* = 0.081), but the relative level of circRNA-089763 in the POCD group was significantly higher than in the NPOCD group on the third day after surgery (fold change = 2.41); the difference was statistically significant (*t* = 4.711, *p* < 0.001).

As shown in Table 6, the binary logistic regression analysis used to analyze the independent predictive factors for POCD showed that age, intraoperative blood loss, and the relative circRNA-089763 level at postoperative 3 days were independently associated with POCD (OR: 1.32, 95% CI: 1.114–1.565, *p* = 0.001; OR: 1.017, 95% CI: 1.004–1.03, *p* = 0.011); OR: 2.75, 95% CI: 1.261–5.999, *p* = 0.011).

**TABLE 6:** Results of binary logistic regression analysis.

Parameter	OR	95%CI	*P-*value
Age	1.320	1.114–1.565	0.001
Years of education	0.693	0.453–1.061	0.092
Intraoperative blood loss	1.017	1.004–1.030	0.011
The relative level of circRNA-089763 at postoperative 3 day	2.750	1.261–5.999	0.011

## Discussion

With the rapid development of medical technology, the number of elderly surgical patients is also increasing (Etzioni et al., 2003). At the same time, the number of patients with POCD has gradually increased, and POCD has become a research hot spot in perioperative medicine (Liu and Leung, 2000). POCD refers to reduced learning, memory, attention, executive function, and language fluency after surgery. POCD is different from postoperative delirium (POD), as POD is accompanied by a change in consciousness, while POCD is not (O’Brien et al., 2017). Therefore, the participants were screened for POD according to the standards for POD diagnosis (Confusion Assessment Method for the Intensive Care Unit scale, CAM-ICU), and none of the participants met criteria for POD in our study. At present, the clinical diagnosis of POCD lacks a unified standard; the International Research Center ISPOCD (Moller et al., 1998) approved the “*Z*-score method” as the best way to judge. In this study, five neuropsychological scales were used to evaluate cognitive function in terms of attention, executive ability, memory learning ability, visual spatial function, and language fluency. During the same period, 20 volunteers were recruited as the control group, and cognitive function assessment was completed. As shown in Table 3, in the volunteers’ two VFT tests, the score of the second test was significantly increased compared with that of the first test, possibly because the previous memory made the second vocabulary expression more fluent, which was caused by the learning effect. Therefore, we recruited volunteers to evaluate the neuropsychological scale and used their ratings as the benchmark to exclude the role of learning effect, thus increasing the credibility of the POCD diagnosis. All enrolled patients were assessed by cognitive scales; *Z* scores were calculated, and when at least two scales of *Z* ≥ 1.96, POCD was diagnosed.

There were 22 POCD patients according to the “*Z*-score method.” In this study, in accordance with the concept of enhanced recovery after surgery (ERAS), most patients may be discharged from the hospital on the seventh day after surgery. Thus, our study evaluated cognitive function on the third day after surgery. This may be different from the test time window of other POCD-related studies, and long-term follow-up of patients is required in future studies to improve cognitive function assessment. In the present study, the 30.56% incidence of POCD on the third day after non-cardiac surgery was lower than in comparable studies (Li et al., 2012; Jiang et al., 2015). Many of the findings in this field vary significantly from study to study, depending on different populations, assessment criteria, intervening measures, surgery types, and other factors. Age was found to be related to the occurrence of POCD in this study, and some studies have also confirmed that age is an independent risk factor for POCD (Griebe et al., 2014). The less years of education and knowledge reserve a patient has, the higher the incidence of POCD (Scott et al., 2017). In the process of surgery, a large amount of blood loss led to circulatory fluctuations and insufficient blood supply to the brain, thereby affecting oxygen supply, brain cell metabolism, and postoperative cognitive function (Zhang et al., 2019). Postoperative pain was related to the occurrence of POCD, and while the relevant mechanism is still unclear at present, it may involve the stress reaction of the body due to pain, inciting a state of anxiety and depression. Some studies have confirmed that effective analgesia can reduce the occurrence of POCD (Wang et al., 2014).

Currently, the occurrence of POCD is related to various peripheral blood biological markers, such as S-100β protein, neuron-specific enolase (NSE), and glial cell line-derived neurotrophic factor (GDNF), but there is still much controversy about the relationship of these markers with POCD (McDonagh et al., 2010; Duan et al., 2018). Therefore, there is still a lack of sensitive and specific biological markers in clinical practice. In the human genome, protein-coding genes account for less than 2%, while non-coding RNAs account for the majority of the genome, playing an important role in the complexity of higher eukaryotes and the disease mechanisms (Mattick, 2001). CircRNA is a class of non-coding RNA with a sponge effect on miRNA, capable of simultaneously adsorbing multiple miRNAs and releasing the inhibitory effect of miRNAs on target genes, thus increasing target gene expression (Hansen et al., 2013). CircRNA is highly stable, conservative, and specific to cell tissues and widely exists in brain, liver, and kidney tissues, as well as plasma exosomes, saliva, and other body fluids. Studies have found that circRNA is involved in the occurrence and development of neurological diseases (Chen et al., 2016), and circRNA is expected to become a new biomarker for neurological diseases, but to our knowledge, there are currently no reports of circRNAs related to POCD. In our previous experiment (Wang et al., 2019), circRNA expression in the plasma exosomes of POCD patients after coronary artery bypass grafting was screened by circRNA gene microarray analysis, and the relative level of circRNA-089763 was found to be significantly increased by qRT-PCR validation. Structural prediction analysis showed that circRNA-089763 could sponge 10 kinds of miRNAs. CircRNA-miRNA-mRNA network analysis also found that circRNA-089763 might bind to miR-6769b-3p, miR-7111-3p, and miR-670-3p at the same time. The three kinds of microRNAs might jointly regulate insulin-like growth factor binding protein-5 (*IGFBP5*) target genes. It has been reported that the *IGFBP5* gene is associated with amyloid-β (Aβ) and cognitive functions (Barucker et al., 2015). CircRNA-089763 also adsorbs miR-6798-3p and miR-3684, which may commonly regulate target genes of tyrosine 3-monooxygenase/tryptophan 5-monooxygenase activation protein gamma genes (YWHAG) and stanniocalcin 1 (STC-1). Research found that STC-1 in the CSF can be used as a biomarker for the differential diagnosis of dementia (Shahim et al., 2017). Ramocki et al. (2010) found that changes in cognitive function and behavioral ability were associated with abnormal expression of the YWHAG gene. Therefore, we speculated that the pathogenesis of POCD might be related to the abnormal circRNA-089763 level caused by perioperative stimuli.

In our previous experiment, we found that it was difficult to collect and extract exosomes due to the need for at least 20 ml of whole blood and experimental equipment, but in clinical work, peripheral blood samples are easy to collect. The mechanism by which POCD occurs after cardiac and non-cardiac surgery is basically the same, but there is a difference in POCD incidence. Exosomes are secreted by most cell types from small membranous vesicles of endocytic origin and can be detected in the blood (Li et al., 2015). Exosomes are present in plasma, and the substances extracted from plasma contain the circRNA-089763 from exosomes. Therefore, this study aimed to investigate whether the level of circRNA-089763 in the plasma (not only exosomes of plasma) of elderly patients undergoing non-cardiac surgery exhibited the same changes. Our results showed that patients who developed POCD also had significantly upregulated circRNA-089763 plasma levels on the third day after surgery, which was similar to the result of our preliminary experiment (Wang et al., 2019). We further analyzed and found that the occurrence of POCD was correlated with the level of plasma circRNA-089763 on the third day after operation. Thus, we have reasons to believe that the relative level of plasma circRNA-089763 is involved in the cognitive function changes. In Netto Martins Back et al.’s (Netto et al., 2018) study, the researchers confirmed that damage to mitochondrial function was associated with POCD. Moreover, the preliminary experiment found that circRNA-089763 was derived from the mitochondrial genome. The damaged mitochondrial function is part of the pathophysiological mechanism of POCD, which will provide a new direction for the study of POCD. Therefore, in non-cardiac surgery, the relative level of plasma circRNA-089763 is increased with POCD, but the mechanism involved remains unclear. It may be related to the effect of circRNA-089763 on IGFBP5, STC, and YWHAG expression by adsorption of the corresponding miRNAs, thereby indirectly regulating cognitive function, a mechanism that needs to be further confirmed in subsequent studies.

Although the mechanism of POCD is still not clear at present, many related studies are in progress. Moreover, a large number of studies have explored how circRNA is involved in the occurrence and development of diseases. In the clinical setting, large sample, multi-center observational studies can be undertaken, and further studies at the gene level can be confirmed in animal and cell-based experiments. It is hoped that this study can provide new ideas and direction for the discussion of biomarkers and their mechanisms in POCD in the future.

## Data Availability Statement

The raw data supporting the conclusions of this article will be made available by the authors, without undue reservation.

## Ethics Statement

The studies involving human participants were reviewed and approved by the protocol was reviewed and approved by the Ethics Committee of Clinical trial in Affiliated Hospital of Southwest Medical University. The patients/participants provided their written informed consent to participate in this study.

## Author Contributions

HZ, FL, WY, MW, and XW conceptualized and designed this study. XZ and MW contributed to data analyses. HZ drafted this manuscript. All authors contributed to interpret the data, revised the manuscript, approved the final content, and read and approved the final manuscript.

## Conflict of Interest

The authors declare that the research was conducted in the absence of any commercial or financial relationships that could be construed as a potential conflict of interest.
